# Determination of Pb^2+^ by Colorimetric Method Based on Catalytic Amplification of Ag Nanoparticles Supported by Covalent Organic Frameworks

**DOI:** 10.3390/nano12162866

**Published:** 2022-08-19

**Authors:** Dongmei Yao, Huiling Bi, Huimin Gong, Hongfang Lai, Sufen Lu

**Affiliations:** School of Chemical and Biological Engineering, Hechi University, Yizhou 546300, China

**Keywords:** AgCOFs, colorimetric method, catalysis, aptamer, Pb^2+^

## Abstract

In this paper, covalent organic frameworks (COFs) are prepared by solvothermal synthesis using 1,3,5-benzenetricarboxaldehyde and benzidine as ligands. Then, using COFs as a template, AgCOFs with high catalytic activity is prepared by in situ loading silver nanoparticles (AgNC) on the surface of COFs by sodium borohydride reduction method. AgCOFs are characterized by TEM, SEM, FTIR and XRD. At the same time, the catalytic ability of AgCOFs for trisodium citrate-AgNO_3_ nanosilver reaction is studied. The results show that AgCOFs can catalyze the reaction of trisodium citrate-AgNO_3_ to generate silver nanoparticles (AgNPs). The solution color of the system gradually changes from colorless to yellow, and the absorbance value increases. Based on the catalytic reaction of AgCOFs and the regulation effect of nucleic acid aptamer reaction on AgCOFs, a new “on–off–on” colorimetric analysis platform is constructed and applied to the detection of trace Pb^2+^ in water samples. This analytical platform is simple, sensitive and selective. Finally, the catalytic mechanism of the system is discussed to verify the feasibility of constructing a colorimetric analysis platform.

## 1. Introduction

Catalysis is one of the important means of signal amplification, and the preparation of high-efficiency and low-consumption catalysts and their application in molecular spectroscopy has always been a research hotspot. Precious metal nanoclusters have the advantages of excellent catalytic performance, adjustable particle size, and good biocompatibility, and are widely used in fluorescence, catalysis, electrochemical sensors and other fields [1,2]. The size of nanoclusters is usually regulated by adding a template, but although the template provides stability, it is usually accompanied by a decrease in the catalytic activity of the nanoclusters [3]. Therefore, choosing a good template can improve the activity of nanoclusters. At present, the templates used for the preparation of noble metal nanoclusters are mostly proteins, polymers, organic salts, etc. [4]. These templates hinder the interaction of reactants with the catalytic surface of catalysts, thereby reducing or even eliminating their catalytic activity [5]. Therefore, it is of great significance to study noble metal nanocluster templates with excellent stability and catalytic activity. Covalent organic frameworks (COFs), as an emerging class of nanozymes, have excellent catalytic properties and good thermal and chemical stability, and have been regarded as one of the most attractive materials since their discovery in 2005 [6]. The catalysts prepared by the post-functionalization reaction of COFs have achieved great success [7], and the combination of COFs and metal ions can endow COFs with synergistic catalytic properties, thereby improving their catalytic activity [8]. At present, the doping of noble metal nanoparticles in COFs has been applied in the fields of chemical sensing, fluorescence detection and other analytical chemistry [9]. However, most of these applications are in the field of separation analysis, and even if they are used as catalysts, the excellent potential of their catalytic performance in quantitative analysis has not been effectively exploited. Moreover, these methods simply dope COFs and noble metal nanoparticles together, and there are few reports on the preparation of noble metal nanoclusters using COFs as templates. Wang et al. [10] prepared a Pd cluster-loaded COF and used it to catalyze a novel stable Au@NiP nano-reaction with strong resonant Rayleigh scattering (RRS) peaks to realize the RRS analysis of melamine. The linear range of the method was 0.0025–0.04 nmol/L, the detection limit (DL) was 1.96 × 10^−4^ nmol/L, and the sensitivity of the method was high. It has been reported that when noble metals are loaded onto COFs with different pore sizes, the addition of noble metal elements increases the catalytic binding sites of COFs, reduces the aggregation of nanoclusters, and greatly improves their catalytic activity compared with conventional COFs. It can be seen that it is feasible to use COFs as templates to prepare noble metal nanoclusters, and the catalytic activity of the nanoclusters will not be affected by its spatial structure. Based on this, it is of great significance to study the preparation of noble metal nanoparticles supported by covalent organic frameworks and their nanocatalytic quantitative analysis, which plays an important role in improving the sensitivity of analytical methods.

In addition to sensitivity, method selectivity is also important. Based on the specific recognition properties of aptamers for target molecules, it is often used to improve the selectivity of methods. When aptamers bind to target molecules, they can fold into three-dimensional structures and attach specifically and tightly to the surface of their corresponding target molecules [11]. The emergence of aptamers has opened a new research avenue in the field of biochemistry, and they have shown good application prospects in the detection of sensors, small organic molecules and metal ions [12,13]. Based on the catalytic amplification of AgCOFs, the combination of nanocatalytic technology with aptamer-specific reactions has made outstanding contributions to the improvement of method selectivity. Wen et al. [14] prepared an AuCOF catalyst. Based on the regulation of AuCOF catalytic ability by aptamers, an RRS analysis method was established for the detection of Co^2+^ in water with a detection limit of 0.02 nmol/L. The method is sensitive and specific. There are also a few reports on the quantitative analysis of catalytic amplification and aptamer reactions based on COF-supported nanoclusters, but they have not been effectively developed and have great potential for development. Yao et al. [15] reported a COF-supported gold nanoclusters (AuCOF) material. Using the synergistic catalysis of COF and gold nanoclusters and the regulation of aptamers on the catalytic performance of AuCOF, a SERS/RRS dual-mode analysis platform was established for the detection of adenosine triphosphate. The method has high sensitivity and specificity. At present, the analytical techniques used in these methods are mainly SERS and RRS, and the methods are sensitive, but the analytical instruments are relatively expensive, and the cost is high. Therefore, the development of sensitive, selective and low-cost analytical platforms can be considered as a new research direction.

The detection of trace elements in water is one of the standards to measure the quality of water in the environment. There are many trace elements in water, such as zinc, copper, calcium, lead and so on. Pb^2+^ is a trace element with toxic effects in water quality, and it is also recognized as one of the most toxic metal ions, which brings great harm to people’s life and health. Therefore, it is necessary to develop a simple, sensitive and selective analytical platform for the detection of Pb^2+^. At present, the detection methods of Pb^2+^ include electrochemical method [16,17], colorimetric method [18], surface-enhanced Raman scattering (SERS) spectroscopy [19], nuclear magnetic resonance sensor [20], fluorescence method [21], chemiluminescence method [22], resonance light scattering method [23], etc. Among them, the electrochemical method is simple and sensitive, but there are many factors affecting electrode modification, which is not conducive to control and real-time monitoring experiments. The SERS method is sensitive, but the instrument is expensive, and the cost is high. Fewer chemical reactions are used for chemiluminescence, which limits the application of chemiluminescence method. The colorimetric method is easy to operate and low in cost, which is conducive to large-scale promotion. Wang et al. [24] prepared a highly catalytic and stable COF by the polycondensation of trialdehyde-based phloroglucinol and p-phenylenediamine, and doped Pd into COF to prepare COFPd. COFPd was used to catalyze the reduction of Ni(II) by sodium hypophosphite to form a Ni-P alloy, and finally, the spectrophotometric detection of Pb^2+^ was realized. The method combines COFPd, nanocatalysis and aptamer reaction, and the method is simple, sensitive and selective. In this paper, based on the catalytic amplification of Ag nanoparticles supported by covalent organic frameworks, and the catalytic activity of AgCOFs can be regulated by aptamers, a colorimetric sensor was constructed for the detection of Pb^2+^. This method is used to detect the content of Pb^2+^ in water quality, the recovery rate of the method is between 93.9–102.5%, and the results are satisfactory.

## 2. Materials and Methods

### 2.1. Reagents and Instruments

Nucleic acid aptamer, sequence: 5’-GGTTGGTGTGGTTGG-3’ was purchased from Sangon Bioengineering (Shanghai) Co., Ltd., Shanghai, China. 1,3,5-benzenetricarboxaldehyde was purchased from Jiangsu Yangnong Chemical Group Co., Ltd., Jiangsu, China. Benzidine was purchased from McLean research center, Shanghai, China. Glacial acetic acid was purchased from Tianjin Beichen Fangzheng reagent factory, Tianjin, China. N, N-Dimethylidene (DMF) and tetrahydrofuran (THF) were purchased from Tianjin Zhiyuan Chemical Reagent Co., Ltd., Tianjin, China. AgNO_3_ and trisodium citrate (TC) were purchased from Tianjin Guangfu Institute of Fine Chemicals, Tianjin, China. The reagents used in the experiments are all analytical grade, and the water used is ultrapure water.

An 8453 UV-Vis Spectrophotometer (Agilent Technologies Co., Ltd., Santa Clara, CA, USA), MiniFlex 600 X-ray Powder Diffractometer (Rigaku Corporation, Tokyo, Japan), NICOLET 6700 Fourier Transform Infrared Spectrometer (Thermo Fisher Scientific, Massachusetts, MA, USA) and Transmission Electron Microscope (JEOL Ltd., Tokyo, Japan) were used. 

### 2.2. Preparation of COF

In a clean glass bottle, add 48 mg 1,3,5-benzenetricarboxaldehyde, 3 mL 1,4-dioxane, 48 mg benzidine, dissolve by ultrasonic to obtain a brown solution, slowly drop 0.6 mL 3 mol/L glacial acetic acid, after generating a yellow-green precipitate, seal it well, and place the sample in an oven to dry at 120 °C for 3 days. Then, centrifuge it (12,000 r/min, 10 min) and wash three times with DMF (10 mL) and THF (10 mL), respectively, and finally, vacuum freeze-dried for 24 hours to obtain a yellow solid, which is COF.

### 2.3. Preparation of AgCOF

Weigh 10 mg of COF and dissolve it in 10 mL of water by ultrasonic. Under the condition of magnetic stirring, add 208 μL of 10 mmol/L AgNO_3_, 0.8 mL 0.01 g/mL trisodium citrate, and then add 0.45 mL of 0.4 mg/mL NaBH_4_ dropwise. Continue stirring for 15 min to obtain a yellow solution which is AgCOF with a concentration of 0.88 mg/mL (calculated as AgCOF).

### 2.4. Experimental Method

Add 15 μL of 0.1 μmol/L Pb^2+^ aptamer, 150 μL of 0.88 mg/mL AgCOF, and a certain amount of 0.01 μmol/L Pb^2+^ into a 5 mL graduated test tube, and mix thoroughly. After standing for 15 min, add 130 μL 0.01 mol/L AgNO_3_, 100 μL 0.1 mol/L trisodium citrate, and make up to 1.5 mL. After reacting in a water bath at 85 °C for 15 min, terminate the reaction by cooling with ice water. Finally, the UV-Vis absorption signal (A) of the solution is measured, and the blank signal (A_0_) without Pb^2+^ is used to calculate ΔA = A − A_0_.

## 3. Results and Discussion

### 3.1. Experimental Principle

**Figure 1:**
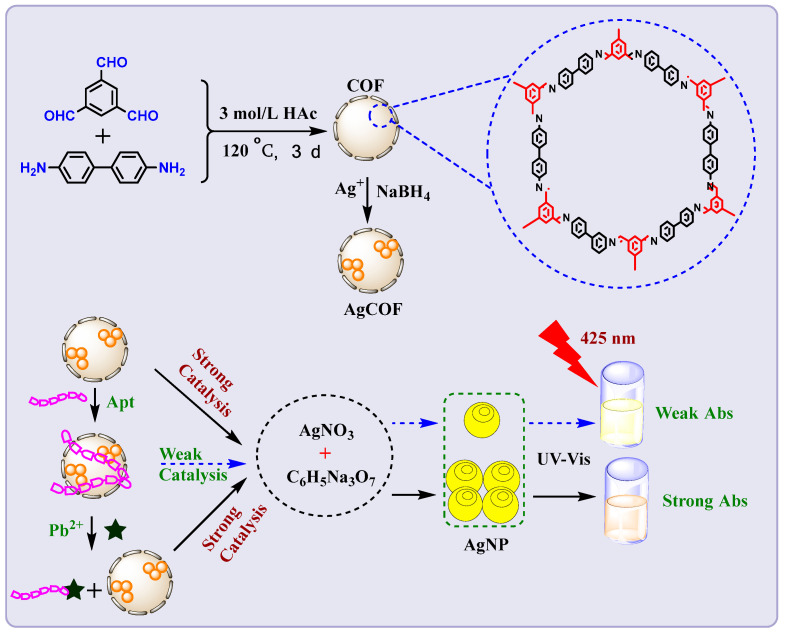
Schematic diagram of the preparation of AgCOF and its catalytic amplified colorimetric determination of Pb^2+.^

COF is a material with a porous framework structure connected by covalent bonds. Metal nanoparticles are loaded in COF, and the size of metal nanoparticles can be controlled by using its pores, which can enhance the dispersibility of metal particles, thereby improving the active sites of COF. In this paper, AgCOFs are prepared by reducing Ag^+^ with sodium borohydride to form Ag nanoparticles which were loaded onto COF. The coordination between Ag atoms and the COF framework can make the AgNCs located in the COF channels more stable, and the active sites located in the channels are more accessible to the reaction substrate. The AgNO_3_–trisodium citrate system hardly reacts under the condition of a high-temperature water bath. AgCOF can quickly catalyze the reaction of the system to generate Ag nanoparticles, and the products can generate a strong absorption signal. Aptamers can be adsorbed to the surface of AgCOF by electrostatic interaction, reducing the effective contact area between AgCOF and the AgNO_3_–trisodium citrate system. As a result, the catalytic performance of AgCOF is inhibited, resulting in a decrease in the absorbance value of the system. Based on the specific reaction between Pb^2+^ and Pb^2+^ aptamer, Pb^2+^ and aptamer will generate G quadruplex. This makes the aptamer desorb from the surface of AgCOF, and the catalytic ability of AgCOF is restored. Finally, the absorption signal of the system increases, and the absorption signal has a linear relationship with the concentration of Pb^2+^. Based on this, a catalytic amplified colorimetric assay based on covalent organic framework-supported silver nanoparticles for the detection of Pb^2+^ is established (Figure 1).

### 3.2. Ultraviolet-Visible (UV-Vis) Absorption Spectra

**Figure 2:**
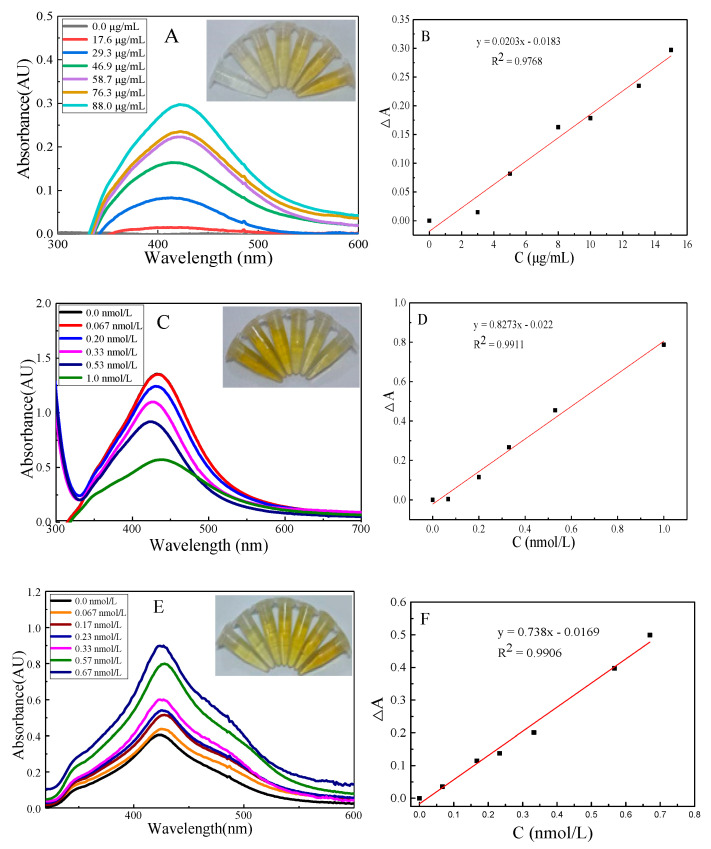
The absorption spectra of the system and their corresponding linear relationship. (**A**,**B**) AgCOF catalytic system: (0, 17.6, 29.3, 46.9, 58.7, 76.3, 88.0) μg/mL AgCOF+ 0.87 mmol/L AgNO_3_+ 6.67 mmol/L TC. (**C**,**D**) Aptamer inhibition system: (0, 0.067, 0.20, 0.33, 0.53, 1.0) nmol/L Pb^2+^ Apt+ 88.0 μg/mL AgCOF+ 0.867 mmol/L AgNO_3_+ 6.67 mmol/L TC. (**E**,**F**) Pb^2+^ determination system: (0, 0.067, 0.17, 0.23, 0.33, 0.57, 0.67) nmol/L Pb^2+^ + 1.0 nmol/L Pb^2+^ Apt + 88.0 μg/mL AgCOF + 0.867 mmol/L AgNO_3_ + 6.67 mmol/L TC.

Under the selected conditions, the AgNO_3_–trisodium citrate system hardly reacts. When AgCOF is added to the nanoreaction system, the oxidant AgNO_3_ and the reducing agent trisodium citrate are adsorbed on the surface of AgCOF, and Ag^+^ is reduced to a small amount of Ag^0^ by trisodium citrate. The surface electrons on Ag^0^ accelerate the redox electron transfer to form AgNPs. The catalytic effect is enhanced with the increase in AgCOF concentration, resulting in the generation of more product AgNPs. With the increase in AgCOF concentration, the color of the catalytic system solution gradually deepened, from colorless to yellow, and the absorption signal gradually increased (Figure 2A,B). When Pb^2+^ aptamer is added, Pb^2+^ aptamer is wrapped on the surface of AgCOF to form AgCOF/Pb–Apt complex. They isolate AgCOF from the catalytic reaction, so that the catalytic activity of AgCOF is inhibited, the absorption signal of the system decreases, and the color of the solution gradually changes from yellow to pale yellow (Figure 2C,D). When the aptamer concentration increases to a certain extent, a slight red shift occurs in the absorption spectra, which might be caused by the formation of AgCOF/Pb-Apt complexes. In the presence of Pb^2+^, due to the specific conjugation of Pb^2+^ to the aptamer, a stable G-quadruplex structure is formed [25]. AgCOF change from a complex to a free state, the catalytic activity is restored, the absorption signal of the system gradually increases with the increase in Pb^2+^ concentration, and the color of the solution gradually changes from light yellow to yellow (Figure 2E,F).

### 3.3. Spectral Characterization

**Figure 3:**
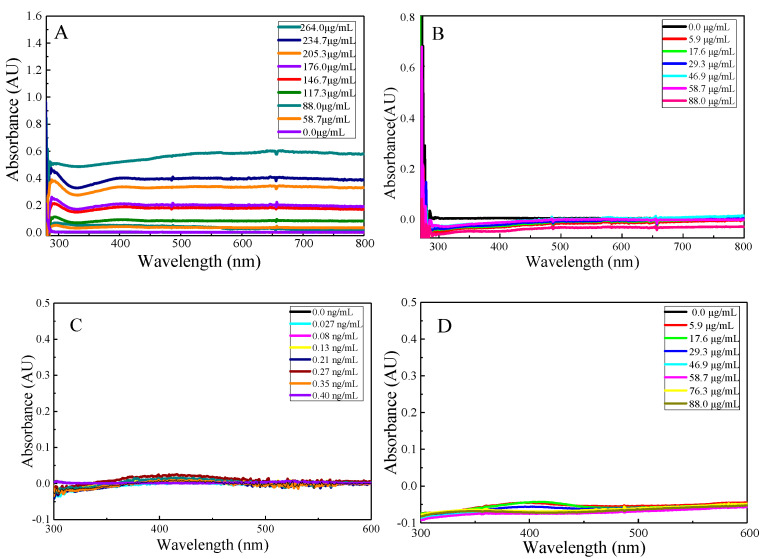
Spectral Characterization. (**A**) UV-Vis absorption spectra of AgCOF at high concentration: (0, 58.7, 88.0, 117.3, 146.7, 176.0, 205.3, 34.7, 264.0) μg/mL AgCOF. (**B**) UV-Vis absorption spectra of AgCOF at low concentration: (0, 5.9, 17.6, 29.3, 46.9, 58.7, 88.0) μg/mL AgCOF. (**C**) UV-Vis absorption spectra of NaBH_4_-AgNO_3_-TC system: (0, 0.027, 0.08, 0.13, 0.21, 0.27, 0.35, 0.4) ng/mL NaBH_4_+ 0.87 mmol/L AgNO_3_+ 6.67 mmol/L TC. (**D**) UV-Vis absorption spectra of AgCOF-AgNO_3_ system: (0, 5.9, 17.6, 29.3, 46.9, 58.7, 76.3, 88.0) μg/mL AgCOF + AgNO_3_, 85 °C.

In this paper, the UV-Vis absorption spectra of AgCOF are studied. As can be seen from Figure 3A that AgCOF has a weak absorption peak at 286 nm, and the absorbance at 286 nm gradually increases with the increase in AgCOF concentration. The absorption peak wavelengths of AgCOF and AgNP are inconsistent, which indicate that Ag in AgCOF does not exist in the form of AgNP alone, but they are loaded on the surface of COF with COF as a template to form an AgCOF composite. When the AgCOF concentration is diluted to 88.0 μg/mL, there is no obvious absorption peak of AgCOF (Figure 3B), which indicates that the absorption of AgCOF had no effect on the catalytic system. Based on the strong reducibility of NaBH4, the effect of NaBH_4_ concentration on the AgCOF catalytic system is investigated, and the results show that NaBH_4_ had no effect on the system at a certain concentration (Figure 3C). The absorption spectrum of the AgCOF–AgNO_3_ system under the condition of 85 °C water bath is shown in Figure 3D. It can be seen from the results that there is no obvious absorption peak in this system, which indicates that in the absence of trisodium citrate, AgNO_3_ will not be reduced to AgNP by AgCOF.

### 3.4. Fourier Transform Infrared Spectra (FTIR) and X-ray Powder Diffraction (XRD)

**Figure 4:**
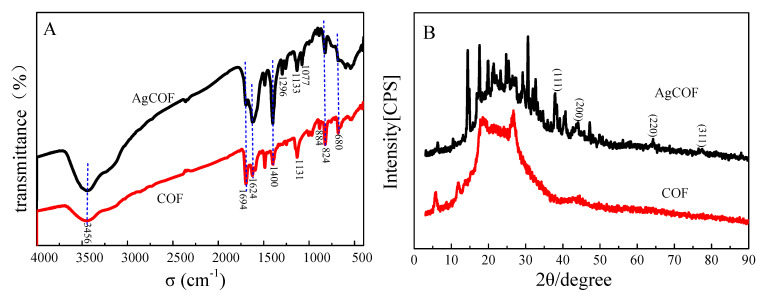
(**A**) FTIR spectra of COF and AgCOF; (**B**) XRD of COF and AgCOF.

To determine whether the loading of Ag nanoparticles affects the framework structure of COFs after the COFs are loaded with Ag nanoparticles, FTIR tests are performed on COFs and AgCOFs, respectively (Figure 4A). It can be seen from the results that the absorption peak of COF at 3456 cm^−1^ is the stretching vibration absorption peak of N-H. There is a sharp absorption peak at 1694 cm^−1^, which is caused by C=N stretching vibration absorption. The absorption peaks at 1624 and 1485 cm^−1^ are the C=C stretching vibration absorption peaks of benzene ring. The absorption peaks at 884 cm^−1^ and 680 cm^−1^ indicate the existence of 1,3,5-substituted phenyl groups, and the absorption peak at 823 cm^−1^ indicates the existence of 1,4-substituted phenyl groups. Thus, the skeleton structure of COF is determined. From the results, we can see that the positions of all absorption peaks do not change significantly after the Ag nanoparticles are loaded on the covalent organic framework (COF). From the results, we can see that the positions of all absorption peaks do not change significantly after the Ag nanoparticles are loaded on the covalent organic framework (COF). This indicates that the loading of Ag nanoparticles does not affect the stability of the COF framework. After the Ag nanoparticles are loaded, a new absorption peak appeared in AgCOF at 1077 cm^−1^, which may be caused by the coordination vibration of Ag-N.

To further determine the loading of Ag nanoparticles on COF, X-ray powder diffraction analysis of COF and AgCOF is carried out. It can be seen from Figure 4B that both COF and AgCOF have an XRD peak at 5°, which is the characteristic diffraction peak of COF [15]. Compare with COF, AgCOF has obvious diffraction peaks at 38.12°, 44.03°, 64.2°, and a weak diffraction peak at 77.14°. These four diffraction peaks correspond to the (111), (200), (220), and (311) crystal planes of Ag, respectively, indicating the existence of Ag nanoparticles.

### 3.5. Transmission Electron Microscopy (TEM) and Energy Dispersion Spectroscopy (EDS)

**Figure 5:**
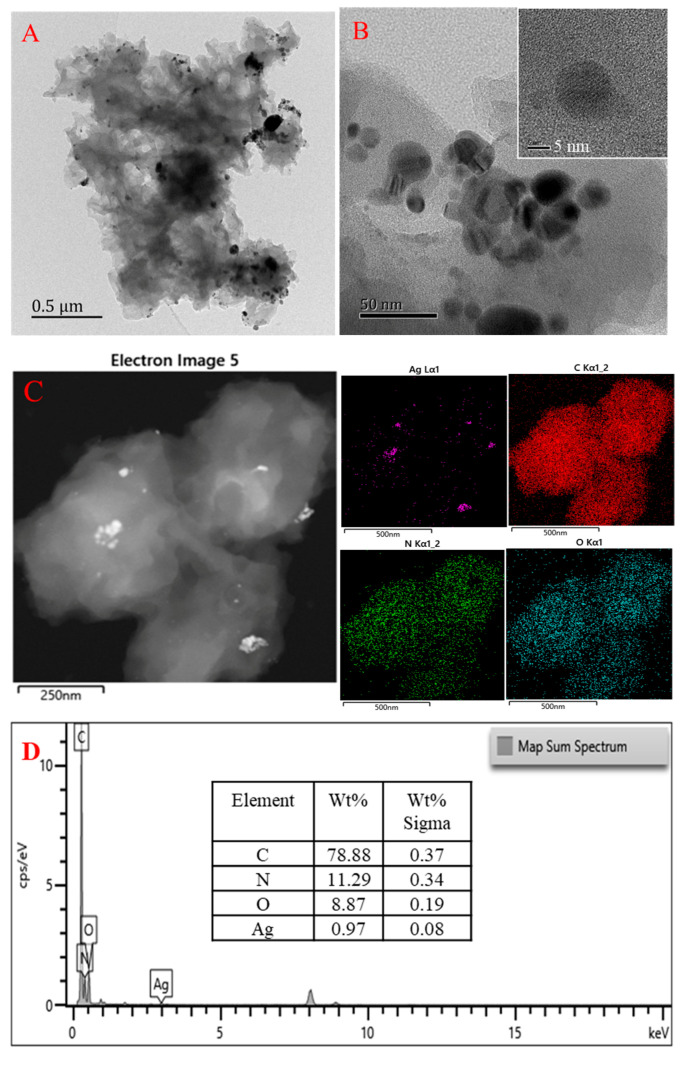

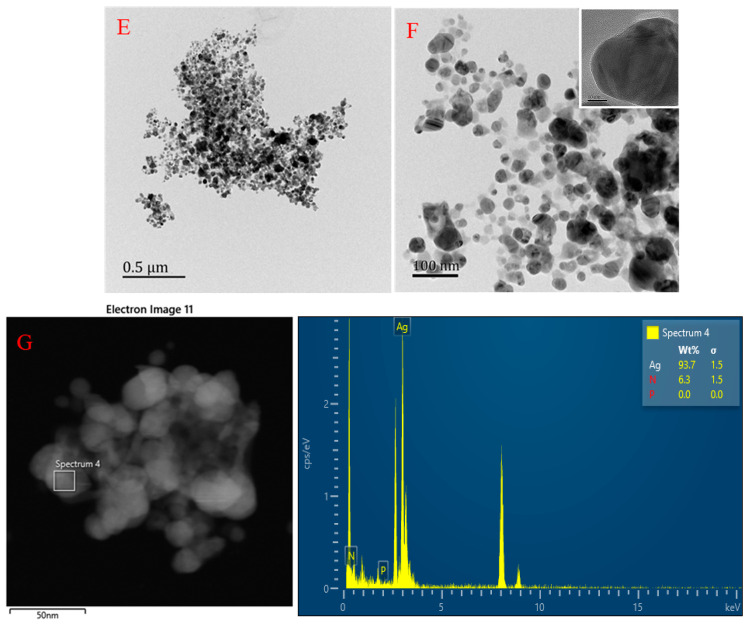
TEM and EDS. (**A**,**B**) TEM of AgCOF; (**C**,**D**) mapping EDS of AgCOF; (**E**,**F**) TEM of AgNP; (**G**) EDS of AgNP.

To determine the morphology and element distribution of COF and AgCOF, TEM and EDS tests are carried out on the system. The samples are prepared according to the experimental method, and the prepared sample solution is diluted with anhydrous ethanol to a certain number, and then the morphology and element distribution of the samples are recorded by transmission electron microscope. It can be seen from Figure 5A that AgCOF is a porous structure, the pores are spherical, and Ag is distributed on the surface of the COF in the form of clusters. After magnifying the AgCOF (Figure 5B), it is found that the Ag nanoparticles are spherical, and most of the particles are within 10 nm in size (the inset of Figure 5B), but the particle size distribution is not uniform enough. As can be seen from the mapping EDS image of AgCOF (Figure 5C) that AgCOF is mainly composed of four elements: C, N, O and Ag. The map sum spectrum of AgCOF (Figure 5D) shows that the weight percentages of C, N, O and Ag elements in AgCOF are 78.88%, 11.29%, 8.87% and 0.97%, respectively, and the content of Ag element is relatively small. However, it also confirmed that Ag nanoparticles are successfully loaded onto the COF surface. To analyze the morphology of the AgNPs generated by the reaction, the AgNP sample solution is prepared according to the experimental method, and its morphology and element distribution are recorded under a transmission electron microscope. It can be seen from Figure 5E,F that the AgNPs are spherical, with an average particle size of about 60 nm. Most of the particles are dispersed in the solution, but some AgNPs are also agglomerated. The lattice fringes of AgNPs can be clearly seen from the inset of Figure 5F. It can be seen in the EDS image of AgNP (Figure 5G) that the main element in AgNP is Ag, accounting for 93.7% by weight.

### 3.6. Particle Size Analysis

**Figure 6:**
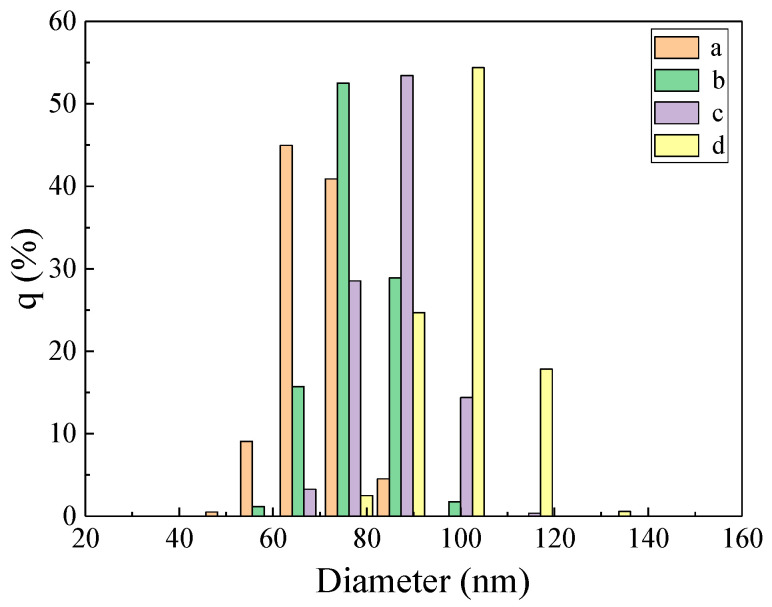
Particle size distribution. (a) 1.0 nmol/L Pb^2+^ Apt + 88.0 μg/mL AgCOF + 0.87 mmol/L AgNO_3_ + 6.67 mmol/L C_6_H_5_Na_3_O_7_; (b) a + 0.067 nmol/L Pb^2+^; (c) a + 0.23 nmol/L Pb^2+^; (d) a + 0.57 nmol/L Pb^2+^_._

To assist in analyzing the particle size distribution of AgNPs in the system, the prepared AgNPs are recorded with a particle size analyzer (Figure 6). It can be seen from the results that the average particle size of AgNPs in the system is 66, 73, 79, and 92 nm, respectively. The measurement results are basically consistent with the detection results of TEM. With the increase in Pb^2+^ concentration, the average particle size of the system increases gradually. This may be due to the gradual increase in the generated AgNPs, and some AgNPs agglomerated to form larger AgNPs.

### 3.7. Analysis of Pore Characteristics of COF

**Figure 7:**
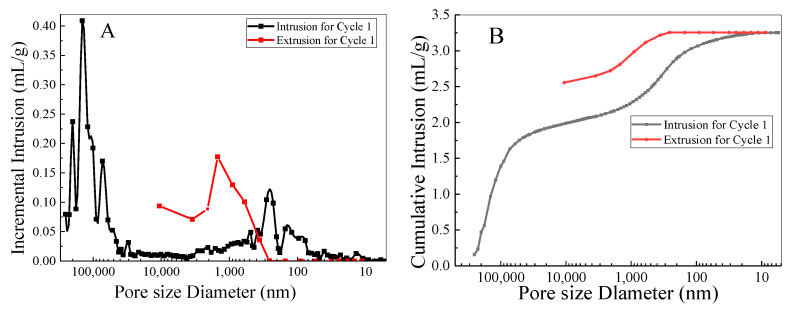
Pore size distribution (**A**) and cumulative pore volume (**B**) of COF.

The pore characteristics of COF are analyzed by the mercury intrusion method. Figure 7 reveals the pore size distribution (A) and cumulative pore volume (B) of the COFs. It can be seen from the results in Figure 7A that the main pores of COF are mesopores (pore size range is 100.0–10.0 μm) and small pores (pore size range is 10.0–0.1 μm). The average pore diameter 4 V/A of the COF is 380.02 nm, the median pore diameter V is 72.94 μm, and the median pore diameter A is 49.15 nm. The pore size range is 350 μm–5 nm. From the results in Figure 7B, it can be seen that the pore size of the cumulative pore volume of COF enters a flat stage after reaching 50 μm and continues to increase after the pore size reaches 1000 nm. Finally, the porosity of COF can be calculated to be 78.66% according to the volume of mercury pressed into the sample and the volume of the sample. Combined with the porosity and pore characteristics of COF, the pore structure of COF is beneficial to the loading of Ag nanoparticles.

### 3.8. X-ray Photoelectron Spectroscopy (XPS) Spectra

**Figure 8:**
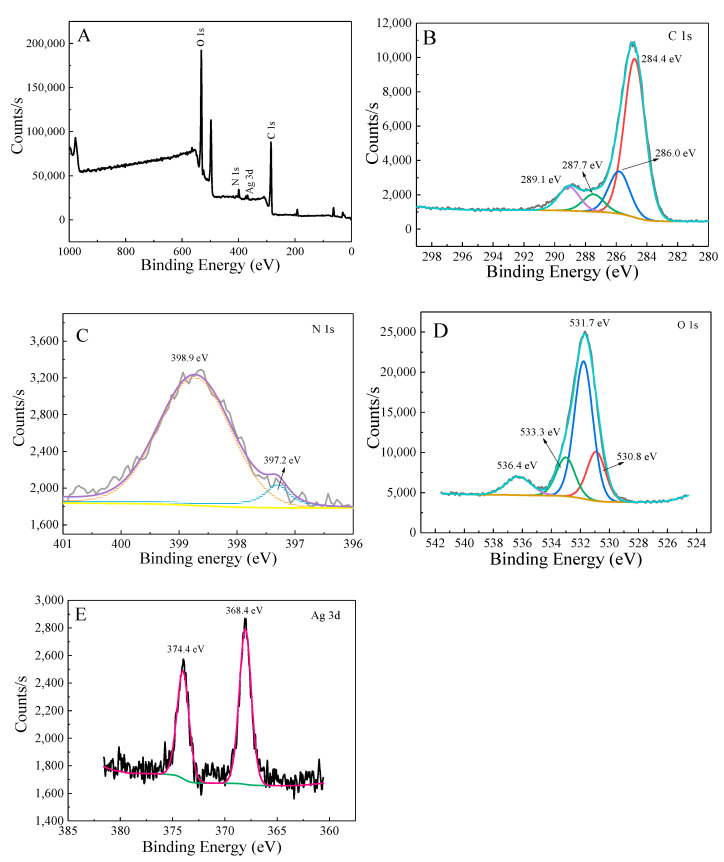
XPS spectra of AgCOF. (**A**) Survey, (**B**) C 1s, (**C**) N 1s, (**D**) O 1s, and (**E**) Ag 3d.

The elemental composition and existing state of AgCOF are analyzed by XPS, and the results are shown in Figure 8. It can be seen from the analysis in Figure 8A that there is a C 1s peak, N 1s peak, O 1s peak and Ag 3d peak in the survey spectrum. The C 1s spectrum in Figure 8B shows that the main peak generated at 284.4 eV is assigned to the C=C bond, while the peaks at 286.6 eV and 287.5 eV are assigned to the C–O and C=O bonds, respectively [26]. The XPS spectrum of N element (Figure 8C) has two peaks, the main peak at 398.8 eV is attributed to the triazine ring (C-N=C) [27], and the peak at 397.3 eV is attributed to the N=Ag bond. The O 1s spectrum in Figure 8D shows that the main peak generated at 531.7 eV is assigned to C=O bonds, and the peak generated at 536.1 eV is assigned to O-H bonds. Figure 8E shows the XPS peaks of Ag 3d in AgCOF. The two peaks are located at 374.4 eV and 368.4 eV, corresponding to the characteristic peaks of zero-valent Ag, representing Ag 3d3/2 and Ag 3d5/2, respectively [28]. The XPS results show that AgCOF has been successfully prepared, and Ag in AgCOF exists in zero valence.

### 3.9. Catalytic Mechanism of AgCOF

**Figure 9:**
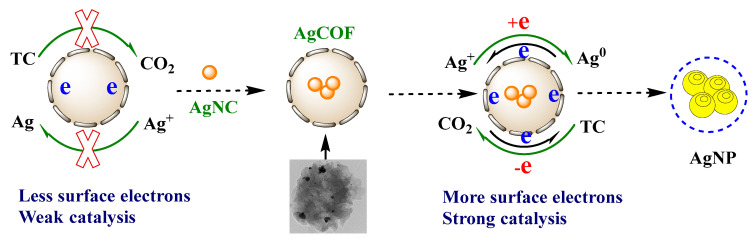
Catalytic mechanism of AgCOF.

Under optimal conditions, the reaction of AgNO_3_–trisodium citrate proceeded very slowly. Meanwhile, COF is not observed to catalyze the reaction, which is mainly attributed to the nano-effect without surface electrons. The surface of COF has large π bonds, but the number of π electrons is relatively small, which is relatively limited in improving the reaction speed of nano-silver reaction. After Ag nanoparticles are loaded, the surface electrons of AgCOF increase, which can rapidly increase the redox electron transfer rate [29]. Due to the abundant pores and C=N bonds on the surface, COFs can provide more active sites. At the same time, the large specific surface area of Ag nanoparticles makes its adsorption effect very strong, and the reactants can be more easily adsorbed on the catalyst surface [30]. After AgNO_3_ and trisodium citrate were adsorbed on the surface of AgCOF, Ag^+^ is first reduced to Ag^0^, and the generated Ag^0^ further accelerates the redox electron transfer to form AgNP (Figure 9). Hence, a faster reaction rate results due to faster charge transfer.

### 3.10. Conditional Optimization 

**Figure 10:**
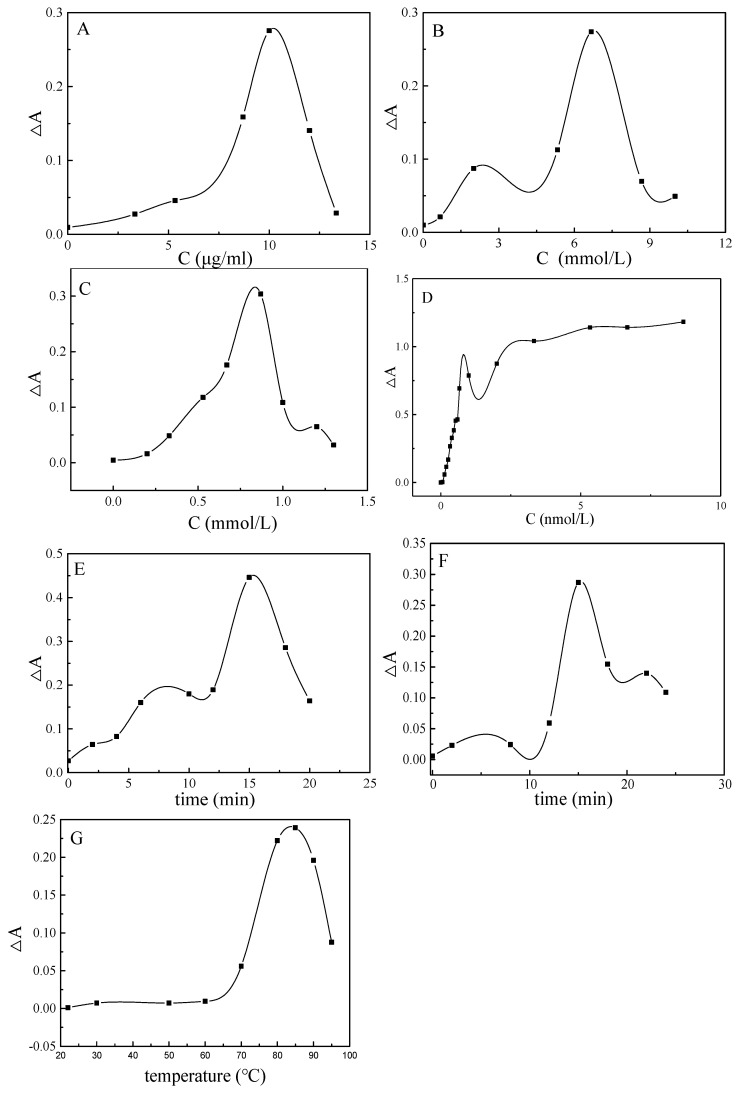
Condition optimization. (**A**): The effect of AgCOF concentration. (**B**): The effect of trisodium citrate concentration. (**C**): The effect of AgNO_3_ concentration. (**D**): The effect of aptamer concentration. (**E**): The effect of standing time. (**F**): The effect of water bath time. (**G**): The effect of water bath temperature.

In this paper, the effects of different conditions on the detection of Pb^2+^ are studied. The results show that when the concentration of AgCOF solution is 88.0 μg/ml (Figure 10A), the concentration of C_6_H_5_Na_3_O_7_ solution is 6.67 mmol/L (Figure 10B), and the concentration of AgNO_3_ solution is 0.87 mmol/L (Figure 10C), the aptamer concentration is 1.0 nmol/L (Figure 10D), the standing time is 15 min (Figure 10E), the water bath time is 15 min (Figure 10F), the water bath temperature is 85 °C (Figure 10G), and the system has maximum ΔA. Therefore, the above parameters are selected as the optimal reaction conditions. Within a certain concentration range, when the concentration of the catalyst AgCOF increases, the more it contacts with the reactants, the more intermediates are produced per unit time, the more the final product AgNP is obtained, and the ΔA increases. However, when the concentration of the catalyst is too large, the catalyst tends to aggregate and settle, thereby reducing the total specific surface area of the catalyst. This will reduce the effective contact area between the catalyst AgCOF and the reactants, reduce the catalytic efficiency and, finally, reduce the ΔA. Trisodium citrate is a reducing agent, and as its concentration increases, the amount of reduced AgNO_3_ increases, the generated product AgNP increases, and ΔA increases. At the same time, trisodium citrate is also a capping agent. If its concentration is too large, it is difficult for Ag^0^ to generate large particles of AgNP, which will cause a decrease in ΔA. AgNO_3_ is used as the precursor of AgNP. When the concentration of AgNO_3_ is low, less AgNP is generated in the system, and the ΔA of the system is low. When the AgNO_3_ concentration is too large, the generated AgNPs will combine to form large aggregates, which will partially adsorb and cause the system ΔA to decrease. At low aptamer concentrations, the background signal is high, resulting in a low ΔA. When the aptamer concentration reaches a certain value, the ΔA of the system gradually becomes stable. The aptamer is specific to Pb^2+^, and the binding time is too short, which has little effect on the catalyst AgCOF. Therefore, within a certain range, with the increase in the standing time, ΔA gradually increases. However, if the standing time is too long, it is difficult for Pb^2+^ to desorb the aptamer from the surface of AgCOF, which, in turn, reduces ΔA. The reaction of trisodium citrate–AgNO_3_ needs to be carried out at a high temperature. Within a certain range, the higher the temperature and the longer the water bath time, the more Ag nanoparticles are generated and the larger the ΔA of the system. When the temperature and time of the water bath exceed a certain value, the generated Ag nanoparticles will aggregate and settle at the bottom of the test tube, and the ΔA of the system will decrease.

### 3.11. Interference Experiment

In addition to sensitivity, method selectivity is also very important. In order to evaluate the selectivity of the method, the interference of possible ions in the water samples on the detection of Pb^2+^ is investigated. The target samples to be tested in this paper are pond water and surface water. Combined with the possible substances in the water samples and the target Pb^2+^ detected in this paper, common metal ions and anions are selected as the interference objects of the simulation. The effects of common metal ions and some anions coexisting with Pb^2+^ on the selectivity of the system are examined, and the results are shown in Table 1. The results show that when the simulated interfering ions concentration is 10–100 times larger than that of Pb^2+^, the relative errors of the detection results are all within 7%. This shows that some common metal ions and anions have little effect on the detection of 0.33 nmol/L Pb^2+^ in a certain concentration range. When the pH is close to neutral, it has little effect on the system. It can be seen from the results that this method has good selectivity.

### 3.12. Working Curve

In order to realize the determination of Pb^2+^ in water samples, a working curve is established under the selected conditions. The content of Pb^2+^ in water samples is quantitatively determined by establishing a linear relationship between the concentration of Pb^2+^ and ΔA. It can be seen from Figure 2F that there is a good linear relationship between Pb^2+^ concentration and ΔA in the linear range of 0.067–0.67 nmol/L. The working curve equation is ΔA = 0.738 C–0.0169, and the linear correlation coefficient is R^2^ = 0.9906. Based on this, a Pb^2+^ assay method for aptamer-regulated AgCOF catalytic activity is established. Compared with the reported methods (Table 2), this method is simple, sensitive and selective.

### 3.13. Analysis Applications

After the analysis method is established, its practical application is also the focus of investigation. In this paper, the method is applied to the determination of Pb^2+^ in actual water samples. Three types of actual water samples (ultrapure water, pond water, and surface water) are collected and labeled as A, B, and C, respectively. After filtration through a 0.45 μm filter, they are marked as samples to be tested. After a certain number of dilutions, the content of Pb^2+^ is determined according to the experimental method, and the results are shown in Table 3. It can be seen from the results that no Pb^2+^ content is detected in ultrapure water, and the Pb^2+^ content in pond water and surface water are 3.9 and 0.87 μg/L, respectively. Five parallel determinations (single measurement value) are performed for each sample, and their relative standard deviations (RSD) range from 5.3% to 7.5%. Then, sample spike recovery experiments are carried out. The concentration of standard Pb^2+^ added in the sample (scalar addition) is between 0.033 and 0.2 nmol/L, and the measured total concentrations of Pb^2+^ (spike measurement values) are 0.205, 0.131 and 0.197 nmol/L, respectively. Accordingly, the recovery rate can be calculated to be between 93.9 and 102.5%. According to the national standard of the People’s Republic of China, the content of Pb^2+^ in water quality is less than or equal to 0.01 mg/L. It can be seen from the test results that the Pb^2+^ content in the three water samples tested is less than 3.9 μg/L, which indicates that the Pb^2+^ content in the samples does not exceed the standard.

## 4. Conclusions

In this paper, COF is used as a supporting template to support Ag nanoparticles. The porous structure of COF provides a micro-reaction site for the loading of Ag nanoparticles. Using the synergistic effect of COF and Ag nanoparticles, and the regulation effect of aptamer on the catalytic performance of AgCOF, when Pb^2+^ is added, the catalytic performance of AgCOF is restored based on the specific binding of aptamer and Pb^2+^. Accordingly, a colorimetric method based on catalytic amplification of covalent organic framework-supported Ag nanoparticles is established to detect the content of Pb^2+^ in water. Compare with other analytical methods, the method is simple, selective and sensitive. Finally, the method is applied to the determination of Pb^2+^ in water, and the recovery rate is between 93.9 and 102.5%.

## Figures and Tables

**Table 1:** Effects of Interfering Substances.

Interfering Substances	Difference Multiples	Relative Error (%)	Interfering Substances	Difference Multiples	Relative Error (%)
SO_3_^2−^	100	−0.25%	Al^3+^	100	0.89%
SO_4_^2−^	100	1.9%	Ag^+^	100	0.49%
NO_2_^−^	100	−0.25%	K^+^	100	0.17%
Br^-^	100	0.17%	Bi^3+^	100	6.8%
Se^4+^	10	0.42%	Ca^2+^	100	0.4%
Sb^3+^	10	−5.3%	Fe^3+^	100	0.026%
Zn^2+^	10	5.4%	As^3+^	100	6.7%
Mg^2+^	10	−2.83%	pH 7.0	/	1.9%
Cu^2+^	100	5.3%	pH 6.7	/	−5.8%

**Table 2:** Comparison with the reported Pb^2+^ analysis methods.

Method	Principle	Linear Range	Detection Limit	Comments	Reference
electrochemical method	Combining the capture ability of glutathione and the electrocatalytic ability of gold nanoparticles for Pb^2+^, the authors developed a sensor for the analysis of Pb^2+^ in rice phloem fluid.	20–200 nmol/L	0.01 μmol/L	Stable and reproducible	[16]
colorimetric method	Ag/Au nanoparticles were prepared by ascorbic acid for colorimetric determination of Pb^2+^.	3–180 nmol/L	1.4 nmol/L	Simple and responsive	[18]
SERS	Based on the combination of a reproducible silicon nanohybrid substrate and a calibrated internal standard sensing strategy, the authors developed a SERS chip for the detection of Pb^2+^ and Hg^2+^.	100 pmol/L–10 μmol/L	19.8 ppt	Wide linear range and sensitivity	[19]
nuclear magnetic resonance senso	The authors developed quercetin-coated Fe_3_O_4_ nanoparticles as low-field NMR sensors for the detection of Pb^2+^ and Cu^2+^.	4.8×10^−6^–1×10^−4^ mol/L	1.6 μmol/L	Good recovery and high adsorption capacity	[20]
fluorescence method	Using a simple and versatile phospho-perylene modification strategy, the authors developed a G-quadruplex probe with a thrombin-binding aptamer for fluorescent detection of Pb^2+^.	3.2–6.1 μmol/L	24.5 nmol/L	good anisotropy	[21]
chemiluminescence method	Based on its displacement of K^+^ in G-quadruplex deoxyribonucleases, a simple and highly sensitive chemiluminescence method for the detection of Pb^2+^ in biological samples was developed.	0.4–10 nmol/L	0.06 nmol/L	Simple and responsive	[22]
resonance light scattering method	The authors used dithiocarbamate-terminated silver nanoparticles as resonance light scattering probes to detect Pb^2+^ and cysteine simultaneously.	0.01 mmol/L –60 mmol/L	4 nmol/L	Good selectivity and wide linear range	[23]
colorimetric method	Based on the catalytic amplification of signals of Ag nanoparticles supported on covalent organic frameworks, a colorimetric analysis method of aptamer-regulated catalytic signals was established for the detection of Pb^2+^.	0.067–0.67 nmol/L	0.060 nmol/L	High sensitivity and good selectivity	This method

**Table 3:** Results of sample determination.

Water Sample	Single Measurement Value (nmol/L)	Average Value (nmol/L)	RSD (%)	Scalar Addition (nmol/L)	Spike Measurement Value (nmol/L)	Recovery Rate (%)	Sample Content (μg/L)
A	Not detected	/	/	0.2	0.205	102.5	/
B	0.093, 0.096, 0.10, 0.092, 0.099	0.1	7.5	0.033	0.131	93.9	3.9
C	0.07, 0.065, 0.068, 0.062, 0.07	0.07	5.3	0.13	0.197	97.7	0.87

## Data Availability

The data presented in this study are available on request from the corresponding author.
